# Orbital entanglement and CASSCF analysis of the Ru–NO bond in a Ruthenium nitrosyl complex

**DOI:** 10.1039/c4cp05278a

**Published:** 2015-03-13

**Authors:** Leon Freitag, Stefan Knecht, Sebastian F. Keller, Mickaël G. Delcey, Francesco Aquilante, Thomas Bondo Pedersen, Roland Lindh, Markus Reiher, Leticia González

**Affiliations:** a Institut für theoretische Chemie , Universität Wien , Währinger Str. 17 , 1090 Vienna , Austria . Email: leticia.gonzalez@univie.ac.at; b ETH Zürich , Laboratory of Physical Chemistry , Vladimir-Prelog-Weg 2 , 8093 Zürich , Switzerland . Email: markus.reiher@phys.chem.ethz.ch; c Department of Chemistry – Ångström , The Theoretical Chemistry Programme , Uppsala University , Box 518 , 751 20 Uppsala , Sweden; d Dipartimento di Chimica “G. Ciamician” , Università di Bologna , V. F. Selmi 2 , 40126 Bologna , Italy; e Centre for Theoretical and Computational Chemistry , Department of Chemistry , University of Oslo , P.O. Box 1033 Blindern , 0315 Oslo , Norway; f Uppsala Center for Computational Chemistry - UC3 , Uppsala University , Box 518 , 751 20 Uppsala , Sweden

## Abstract

Complete active space self-consistent field (CASSCF) wavefunctions and an orbital entanglement analysis obtained from a density-matrix renormalisation group (DMRG) calculation are used to understand the electronic structure, and, in particular, the Ru–NO bond of a Ru nitrosyl complex. Based on the configurations and orbital occupation numbers obtained for the CASSCF wavefunction and on the orbital entropy measurements evaluated for the DMRG wavefunction, we unravel electron correlation effects in the Ru coordination sphere of the complex. It is shown that Ru–NO π bonds show static and dynamic correlation, while other Ru–ligand bonds feature predominantly dynamic correlation. The presence of static correlation requires the use of multiconfigurational methods to describe the Ru–NO bond. Subsequently, the CASSCF wavefunction is analysed in terms of configuration state functions based on localised orbitals. The analysis of the wavefunctions in the electronic singlet ground state and the first triplet state provides a picture of the Ru–NO moiety beyond the standard representation based on formal oxidation states. A distinct description of the Ru and NO fragments is advocated. The electron configuration of Ru is an equally weighted superposition of Ru^II^ and Ru^III^ configurations, with the Ru^III^ configuration originating from charge donation mostly from Cl ligands. However, and contrary to what is typically assumed, the electronic configuration of the NO ligand is best described as electroneutral.

## Abstract 2

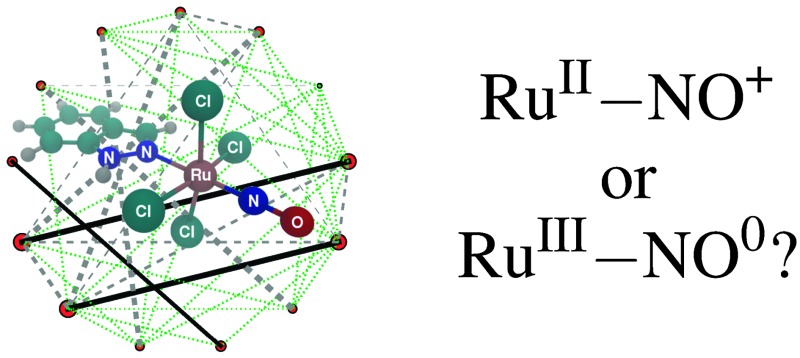
Multiconfigurational wavefunction analysis and entanglement measures based on von Neumann entropy shed light on the electronic structure of a Ru nitrosyl complex, in particular on the Ru–NO bond.

## 1 Introduction

The electronic structure and properties of transition metal nitrosyl complexes have been a subject of interest in inorganic and bioinorganic chemistry for a long time. Nitric oxide (NO) plays a role in neurotransmission, blood pressure control and even control of tumour growth.^1^ A number of transition metal nitrosyl complexes have been employed in photodynamical therapy to deliver targeted NO to biological tissues.^2^ Particularly interesting are ruthenium nitrosyls, since they are postulated to be intermediates^3,4^ in the mechanism of action of novel ruthenium anti-cancer drugs such as NAMI-A^5^ and KP1019.^6^ Understanding the electronic structure of the metal–NO moiety is therefore essential to rationalise the mechanisms for NO delivery from nitrosyl complexes and thus obtain a fundamental understanding of the mode of action of ruthenium anti-cancer drugs.

Furthermore, NO is a well-known non-innocent ligand in coordination chemistry,^7^ which leads to an intricate and ambiguous electronic structure of the transition metal nitrosyls. NO can attach to the metal both in a linear or a bent configuration, depending on the electronic structure of the metal, charge of the free NO and the remaining coordination sphere. The metal-nitrosyl moiety M–NO usually shows strongly delocalised electron density and a strong covalency. Enemark and Feltham^8,9^ have suggested to describe the electronic structure of this moiety as {M(NO)}^*n*^, with *n* being the total number of electrons in the metal d and nitrosyl π* orbitals. Within this framework, however, the electronic character of neither the Ru nor the NO fragment is known, and it is not possible to assign a particular oxidation state to either fragment. For example, it is unclear whether {RuNO}^6^ structures should be treated as Ru^II^–NO^+^ or Ru^III^–NO^0^. A correct description of the oxidation states of the metal and ligands is important, *e.g.*, for the study of the redox processes involved in the metabolism of redox-active anti-cancer drugs.

Attempts to resolve the ambiguity in the metal–NO bond and to assign oxidation states to the metal and the ligands have been carried out in many theoretical, spectroscopic and electrochemical studies.^4,10–14^ It has been largely accepted that most linear {RuNO}^6^ complexes^15^ and many {FeNO}^6^ complexes^16^ are best described as M^II^–NO^+^. Interestingly, and in contrast to this picture, a recent extensive joint experimental and computational study^4^ on one particular {RuNO}^6^ complex concluded that the physical electronic structure of the Ru–NO moiety is better described by Ru^III^–NO^0^, rather than Ru^II^–NO^+^.

The majority of computational studies on transition metal complexes employ density functional theory (DFT).^17^ However, many structures belong to the class of the so-called strongly correlated systems which cannot be described by Kohn–Sham DFT^18^ due to its single Slater determinant approximation. In this respect, Kohn–Sham DFT is conceptually similar to Hartree–Fock theory, which does not incorporate electron correlation (*i.e.* movement of electrons depending on the instantaneous positions of other electrons). Electron correlation can be classified as^19–21^ *dynamic* and *static* (split further into static and nondynamic correlation by some authors^19,21^). Dynamic correlation is responsible for keeping the electrons apart and is found in any quantum mechanical system with more than one electron. Static correlation corresponds to significant mixtures of several electronic configurations and is largely present, *e.g.*, in dissociating molecules and many transition metal compounds. While dynamic correlation can be effectively described by DFT and post-Hartree–Fock methods, such as Møller–Plesset perturbation theory or coupled cluster (CC) methods, the proper description of static correlation requires several Slater determinants or configurations in the *ansatz*. Multiconfigurational methods – such as the complete active space self consistent field (CASSCF),^22^ the restricted active space SCF (RASSCF),^23^ or the density matrix renormalization group (DMRG)^24^ method in its quantum chemical formulation^25^ as well as their corresponding refinements by second-order perturbation theory^23,26,27^ – are then mandatory to describe such systems.

Sizova *et al.* ^11^ were the first to apply a multiconfigurational/valence-bond treatment to a variety of {RuNO}^6^ complexes. Radoń *et al.* ^13^ applied CASSCF localised orbitals and spin densities to analyse the Fe–NO bond in several {FeNO}^7^ complexes, as well as calculated the doublet-quartet energy gap with CASPT2/CASSCF. Recently, we have also used CASPT2/CASSCF to study the electronic structure of another {RuNO}^6^ complex.^14^ Boguslawski *et al.* ^28^ compared the CASSCF spin densities of several Fe–NO complexes with DFT results, deeming both unsatisfactory. This unpleasant situation could only be resolved by calculating the spin density from a large-CAS DMRG wavefunction, in which the complete double-d shell correlation effects could be taken into account.^29^ Double-d shell correlation effects are related to the presence of a large number of electrons in compact d shells, resulting in large radial correlation effects in these shells. The second more diffuse d shell gives additional flexibility to describe such correlation effects, and for many 3d transition metal compounds the second d shell must be present to obtain quantitative accuracy with the CASPT2 method.^30^

The last example illustrates the major limitation of CASSCF – the factorial growth of computational time with the number of correlating electrons and orbitals. Presently, CASSCF calculations are typically limited to active spaces comprising approximately 16 electrons in 16 orbitals. Over the past few decades, several attempts have been made to overcome the CASSCF factorial scaling problem and allow the usage of larger active spaces: the RASSCF method introduces additional subspaces with a restricted number of excitations; the generalised active space (GAS)^31^ concept takes the RAS concept one step further by introducing an arbitrary number of subspaces. RAS and GAS methods allow us to extend the active spaces at the price of having to choose a restriction of the excitation levels; however this degree of freedom makes these methods less straightforward to use than CASSCF. Recently, the GAS method has been combined with Löwdin's partitioning technique^32^ resulting in the SplitGAS method,^33^ which, despite its demonstrated capability to effectively employ up to 10^22^ Slater determinants, still requires algorithmic advances and further development before it can be widely used.

The conceptually different DMRG algorithm employs the reduced density matrix of the system studied to construct and optimise a CAS-like wavefunction, allowing for a polynomial instead of factorial scaling with the number of active orbitals. As a consequence, DMRG allows much larger active spaces than conventional CASSCF, explaining its value for calculations on transition metal complexes dominated by strong static electron correlation^34^ and its remarkable success in transition metal chemistry in recent years.^29,35^

Using the DMRG algorithm, *n*-orbital reduced density matrices are easily obtained from the full density matrix by tracing out contributions from all orbitals in the complementary set of orbitals in the active space. As a consequence, entanglement measures such as the single-orbital entropy^36^ and mutual information^37,38^ calculated from the one-orbital and two-orbital reduced density matrix, respectively, are easily accessible. These orbital-based entanglement measures can be applied to examine the multi-reference character of the electronic wave function. In particular, they can be correlated with the amount of static and dynamic electron correlation in an electronic wavefunction^21^ or exploited to study chemical bonding in molecule formation and dissociation processes.^39^ Thus, they complement the traditional orbital-based correlation measures such as the natural orbital and geminal analysis.^40^

In addition to the factorial scaling with the active space size, the cost of multiconfigurational calculations also scales as O(*n* ^4^) with the number of basis functions. Efficient calculations on large molecules, including large transition metal complexes, cannot be performed unless this scaling is reduced. Using approximate representations of the electron repulsion integrals based on density-fitting (DF) and Cholesky decomposition (CD)^41^ reduces the scaling to cubic, thus enabling multiconfigurational calculations on larger molecules.^42^ Analytical gradients employing combined DF and CD techniques extend the applicability to geometry optimisations.^43,44^

In this work we investigate the electronic structure of the {RuNO}^6^ moiety in the *trans*-[RuCl_4_(NO)(1*H*-indazole)]-complex (RuHIndNO),^45^ which is closely related to the anti-cancer drug KP1019. We perform CASSCF calculations of the S_0_ and T_1_ state of RuHIndNO, describe the electronic structure of the coordination sphere around Ru and analyse the wavefunction in terms of contributions of different configurations and natural orbital occupation numbers. Note that CASPT2/CASSCF singlet-triplet energy splittings have been addressed in ref. 44. Here we also perform orbital entanglement analyses^21,38,39^ based on DMRG calculations of the S_0_ state. We examine different types of electron correlation present in the Ru–ligand bonds and assess the ability of multiconfigurational methods to describe the Ru–NO coordination sphere in RuHIndNO.

To shed more light on the electronic structure of the {RuNO}^6^ complex, we transform the CASSCF wavefunctions of the S_0_ and T_1_ states into a localised orbital basis and analyse the Ru–NO bond and the Ru coordination sphere in terms of configuration state functions (CSFs) based on localised orbitals; we compare the results obtained from the localised orbital analysis to the Mulliken population of Ru d orbitals based on both single-configurational DFT and the CASSCF wavefunction. At the end of our analysis we adress the non-innocence of the NO ligand: in particular, whether NO is to be considered ionic or neutral and the true 4d occupation of the Ru center.

Noting the importance of the double-d shell effects in transition metal compounds,^29,30,46^ we also investigate the double-d-shell effect using entanglement analysis. We perform another DMRG calculation with an active space incorporating another pair of correlating orbitals and a second d shell on Ru.

## 2 Computational details

Geometry optimisation of the lowest singlet (S_0_) and the triplet state (T_1_) has been performed with DFT, using the BP86 functional^47,48^ and the def2-TZVPP basis set.^49,50^ For Ru, the MWB28 effective core potential^51^ (ECP) has been used, and RI-J and MARI-J^52^ approximations were employed for computational efficiency. The triplet geometry has been optimised with the unrestricted Kohn–Sham procedure. The DFT calculations have been performed using the TURBOMOLE 6.5^53^ suite of programs.

Using the optimised S_0_ and T_1_ geometries, single-point CASSCF calculations employing the ANO-RCC-VTZP basis set^54^ and atomic compact Cholesky decomposition (acCD)-based algorithms^41^ with the Cholesky decomposition threshold of 10^–4^ have been performed with the MOLCAS 7.8 program package.^55^ Mulliken population analyses have been done at the CASSCF and PBE^56^ levels of theory with the ANO-RCC-VTZP basis set, as implemented in MOLCAS.^57^

**Fig. 1:**
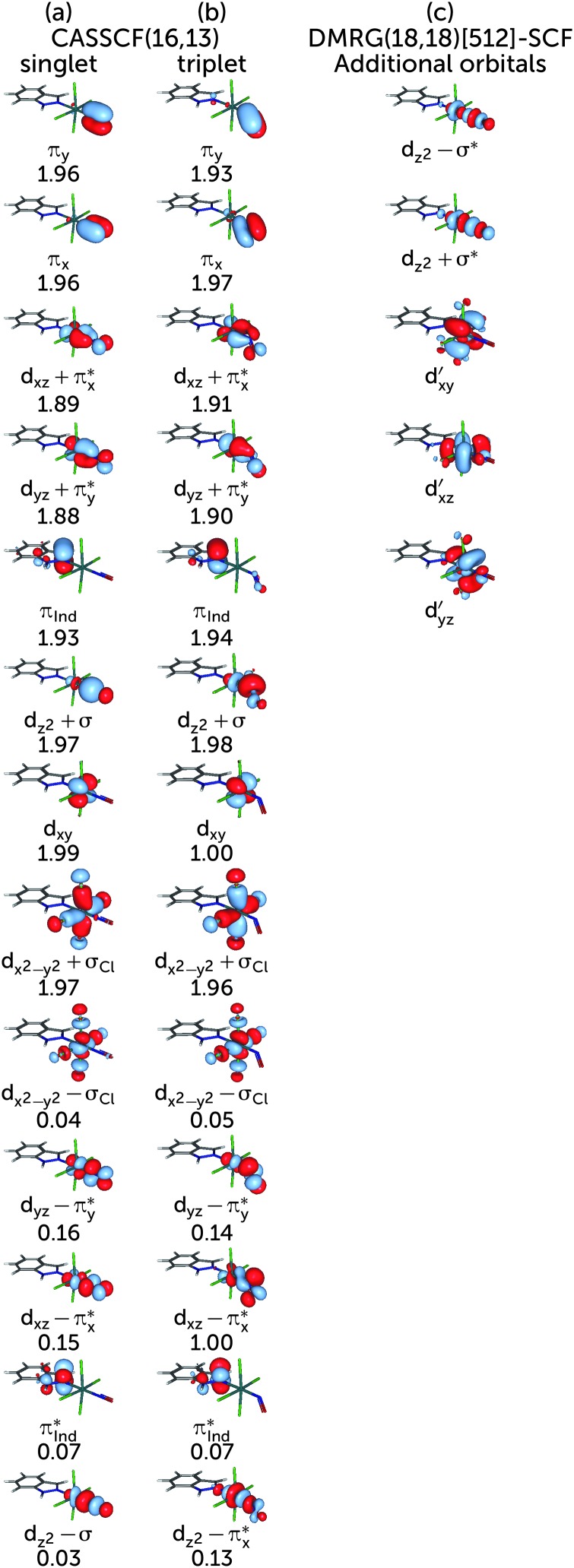
Active space orbitals and their respective occupation numbers used in the optimization of the S_0_ (a) and T_1_ (b) electronic states using CASSCF calculations. Panel (c) shows the additional orbitals used in the DMRG(18,18)[512]-SCF calculation. Double-shell d orbitals are indicated with a prime. The remaining orbitals correspond to those in column (a).

The choice of the CASSCF active space is motivated by its feasibility for a configuration analysis of the Ru coordination sphere. Accordingly, all Ru 4d orbitals and the ligand orbitals interacting with them must be included, resulting in a total active space of 13 orbitals with 16 electrons (denoted (16,13)), including the five Ru 4d orbitals, two pairs of NO π and π* orbitals, one pair of indazole π and π* orbitals, one combination of p orbitals on the Cl atoms (denoted σ_Cl_) as well as the NO σ orbital. The last two orbitals are particularly important because they participate in the covalent bond formed between the metal and the NO and Cl ligands, respectively; accordingly, each of them mixes with the d_*z*^2^_ and the d_*x*^2^–*y*^2^_ orbitals of the Ru atom, respectively. A fair comparison of the CASSCF wavefunction analyses on the S_0_ and T_1_ geometry should be done using the same active spaces in both calculations. For RuHIndNO, this can be only achieved in the S_0_ calculation by a state-average (SA)-CASSCF calculation over the lowest three singlet states. Thus, the T_1_ calculation was similarly averaged over three states, to ensure that the deterioration of the wavefuction quality due to state averaging is similar in both spin states. The resulting orbitals and corresponding natural orbital occupation numbers of the optimised S_0_ and T_1_ geometries are collected in Fig. 1a and b, respectively.

For the entanglement measures, a DMRG-CASCI calculation based on the optimised CASSCF orbitals (*cf.* Fig. 1a) has been performed for the S_0_ state employing the same geometry, active space and basis set as in the CASSCF calculation using the MAQUIS^58,59^ DMRG program, interfaced to the development version of the MOLCAS program package. The number of renormalised active-subsystem states (*m*-value)^58^ is set to 1000. With this value, the DMRG calculation reproduces the absolute energy of the CASSCF calculation up to 10^–6^ a.u., so that the properties of the DMRG wavefunction can be considered identical to those of the CASSCF wavefunction. This calculation will be denoted DMRG(16,13)[1000], using the shorthand notation DMRG(*n*_electrons_,*n*_orbitals_)[*m*]. From the CI-type expansion coefficients of the DMRG wavefunction, a density matrix is constructed, in which environment states can be traced out. These states are states defined on orbitals of the active space that are not considered part of a selected subsystem of orbitals. In the single-orbital case, the selected subsystem consists of only one spatial orbital with four possible states (empty, spin-up, spin-down and doubly-occupied) quantum-mechanically embedded into all other orbitals of the active space. States defined on these complementary orbitals are the environment states traced out in the (then) reduced density matrix. The four eigenvalues of this reduced density matrix, *w*_*α*,*i*_, enter a von Neumann entropy expression, which yields the single-orbital entropy, *s*(1)_*i*_, for a given orbital *i*, which can be understood as a measure of the interaction of one orbital with all other orbitals:1
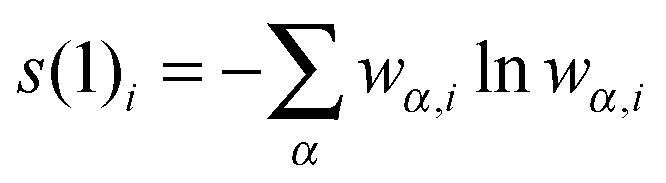
 In the same way, a two-orbital entropy, *s*(2)_*i*,*j*_, can be calculated from the sixteen eigenvalues of the reduced density matrix that is valid for the subsystem consisting of the two selected orbitals *i*,*j*:2
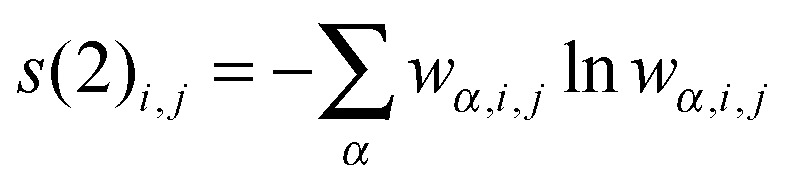
 As this two-orbital entropy still contains single-orbital-entropy contributions, the two single-orbital entropies may be subtracted, which yields the mutual information, *I*_*i*,*j*_, for orbitals *i* and *j*:3
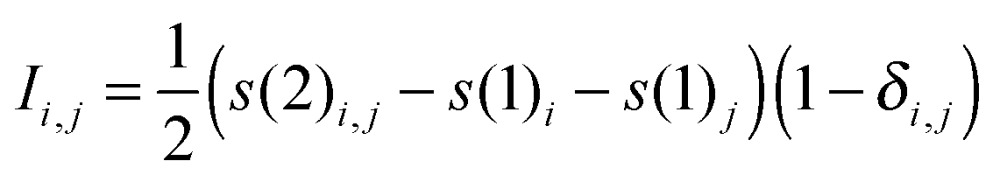
 The central aspect of these measures is that the quantum entanglement of the states defined on one and two orbitals, respectively, has been properly encoded through the eigenvalues of the reduced density matrices.

To evaluate the correlation contribution of additional orbitals, including the second d shell effect, another DMRG calculation and entanglement analysis was performed with a larger active space. The previous (16,13) active space (Fig. 1a) was augmented with another pair of orbitals consisting of Ru d_*z*^2^_ and NO σ* orbitals and the second Ru d_*xy*_, d_*xz*_ and d_*yz*_ shells (*cf.* Fig. 1c). From all orbitals not present in the (16,13) active space, these orbitals were expected to give the largest contribution to the correlation in the Ru coordination sphere. The new active space consists of 18 electrons in 18 orbitals. Since this active space is out of reach for the traditional CASSCF implementation, the orbitals were optimised with the DMRG-SCF approach as implemented in the development version of MOLCAS. We carried out the DMRG(18,18)[512]-SCF orbital optimisation with the smaller ANO-RCC-MB basis set, augmented with an additional d shell on Ru because the ANO-RCC-VTZP basis set yielded additional p shells on N or O atoms of NO instead of the Ru double shell orbitals. The subsequent DMRG-CASCI step, which is based on the DMRG-SCF orbitals, was done increasing the *m*-value back to 1000, to be consistent with the DMRG(16,13)[1000] calculation. The orbital entanglement analysis was carried out for the DMRG-CASCI wavefunction, analogous to what has been explained before.

**Fig. 2:**
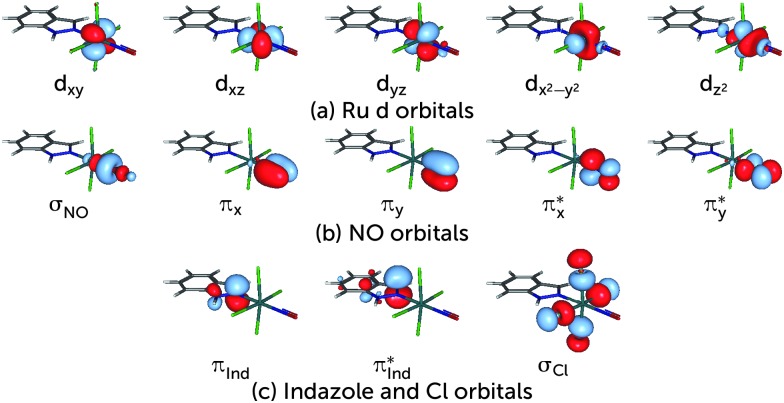
Localised orbitals for the S_0_ structure.

To perform the characterisation of the electronic structure in terms of CSFs based on localised orbitals, all active space orbitals have been localised using the Cholesky algorithm.^60^ As for any rotation among the active orbitals only, this procedure does not change the total energy of the CASSCF wavefunction. The Cholesky localisation yielded orbitals predominantly localised on single atoms, including single p orbitals at the N and O atoms of NO. These were converted into a set of proper π and π* orbitals by forming normalised linear combinations of the form 
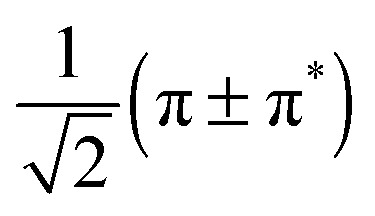
. This procedure yields π and π* orbitals almost exclusively localised on the NO molecule, and together with the other Cholesky orbitals they form the localised active space. The remaining Cholesky orbitals (*cf.* Fig. 2) are d orbitals localised on the metal, the σ orbital localised at the NO molecule and an orbital consisting of the p orbitals of the four Cl ligands. This localised active space is used in the discussion of the electronic structure of the complex.

## 3 Results and discussion

**Fig. 3:**
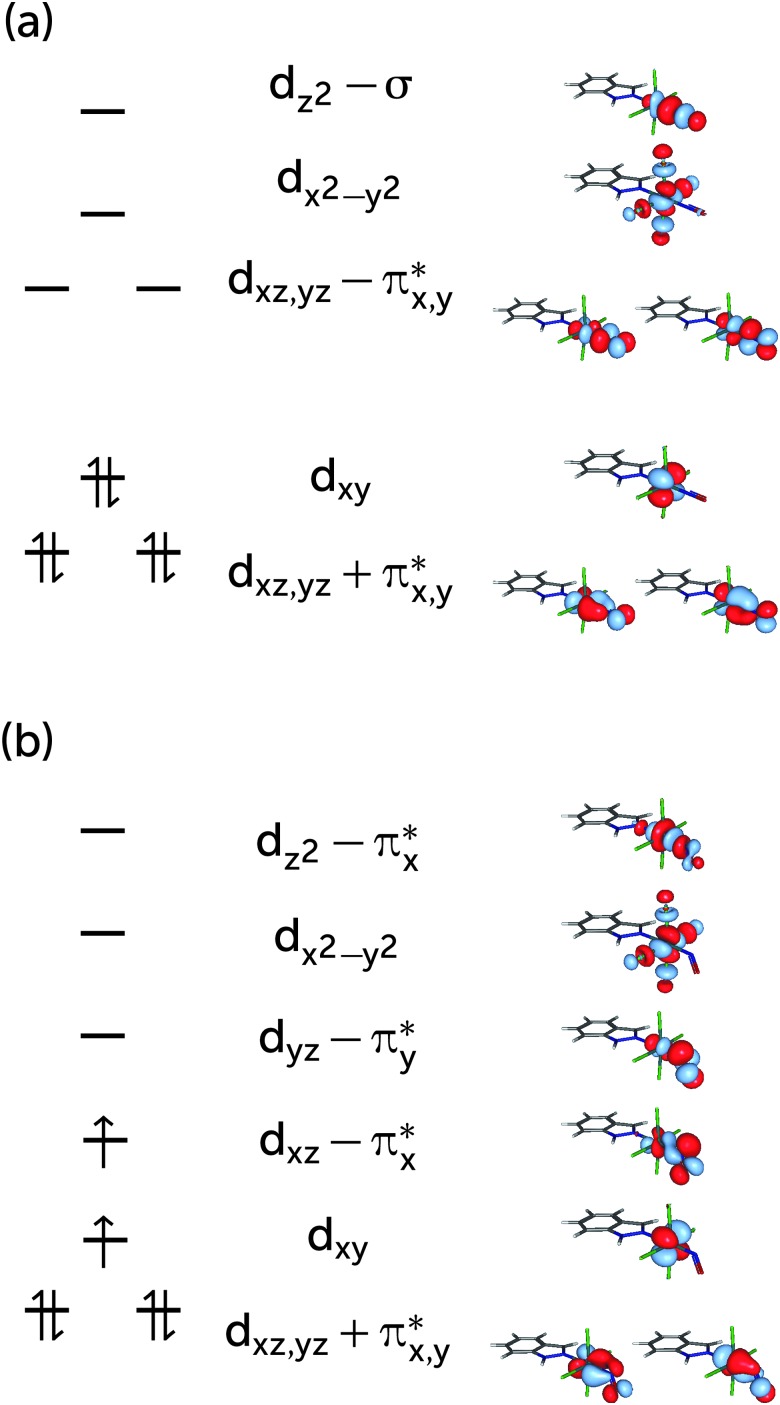
Principal configurations expressed in terms of CASSCF natural orbitals for the S_0_ (a) and the T_1_ (b) state.

Based on the CASSCF wavefunction expressed in natural orbitals (*cf.* Fig. 1a), the singlet, S_0_, and triplet, T_1_, states are predominantly described by the electronic configurations (*cf.* Fig. 1a)|S_0_〉 = |(d_*xz*_ + π_x_*)^2^(d_*yz*_ + π_y_*)^2^(d_*xy*_)^2^(d_*xz*_ – π_x_*)^0^〉|T_1_〉 = |(d_*xz*_ + π_x_*)^2^(d_*yz*_ + π_y_*)^2^(d_*xy*_)^1^(d_*xz*_ – π_x_*)^1^〉respectively (other active orbitals are, respectively, doubly- or unnocupied). Fig. 3 shows a schematic representation of these dominant configurations. In the S_0_ linear structure, the d_*xz*_ and d_*yz*_ orbitals of Ru interact with π* orbitals of NO, forming two bonding and two antibonding orbitals, which are denoted d_*xz*,*yz*_ ± π_*x*,y_*. d_*z*^2^_ of Ru with the σ orbital of NO forms another pair of bonding and antibonding orbitals denoted d_*z*^2^_ ± σ (*cf.* Fig. 1), again indicating a strongly covalent interaction of Ru with NO. The triplet dominant configuration is a d_*xy*_ → d_*xz*_ – π_x_* excitation with respect to |S_0_〉. Since the latter orbital is an antibonding orbital, the Ru–NO bond is weaker in the T_1_ structure than in the S_0_ structure, where the d_*xy*_ orbital is doubly occupied. Indeed, the bond in the triplet geometry (1.838 Å) is longer than that of the singlet (1.718 Å).^44^ Unlike the linear S_0_ structure, the interaction of the d_*z*^2^_ orbital with the π* orbitals of the NO ligand is not symmetry forbidden; therefore a linear combination d_*z*^2^_–π_x_* is formed.

The major configurations discussed above correspond to 77% of the singlet and 78% of the triplet wavefunctions of RuHIndNO. These weights are lower than the typical value of over 90% for a molecule where the ground state is well described with a single configuration. The remaining ≈20% are distributed among many other configurations, each with weights below 3%. One might then be tempted to conclude that a single-configurational description is sufficient in this case, arguing that the remaining wavefunction contributions are negligible or that they arise due to the dynamic correlation of the system, present in every molecule. If that was the case, however, double excitations would dominate the remaining configurations and single excitations would have much less weight due to the Brilloin theorem. Indeed, one can find that the configuration with the second largest weight of (3% and 2%) in the S_0_ and T_1_ wavefunctions, respectively, is a local π → π* double excitation on the indazole ligand; this configuration can be attributed to dynamic correlation between these orbitals. However, a number of single excitations with comparable weight are also present in the wavefunction, for instance, the (d_*xz*_ + π_x_*) → (d_*xz*_ – π_x_*) and (d_*yz*_ + π_y_*) → (d_*yz*_ – π_y_*) excitations in the S_0_ wavefunction, and excitations to the (d_*xz*,*yz*_ – π_*x*,y_*) and d_*z*^2^_ – π_x_* orbitals, in the T_1_ state, which points to the presence of static correlation. For comparison, the former contributions amount to 18% of the S_0_ wavefunction in the related [Ru(PaPy_3_)(NO)]^2+^ complex.^14^

The presence of both static and dynamic correlation in the Ru–NO bond of RuHIndNO is also reflected by the occupation numbers of the orbitals involved in the Ru–NO bonds, which differ significantly from the formal values of 2 (doubly occupied) and 0 (unoccupied). In the S_0_ state these are the (d_*xz*,*yz*_ + π_*x*,y_*) and (d_*xz*,*yz*_ – π_*x*,y_*) orbitals with occupation numbers of 1.89, 1.88, 0.15 and 0.16 respectively (*cf.* Fig. 1). Similar occupation numbers for these orbitals are also found in the T_1_ state, although here the role of the (d_*yz*_ – π_y_*) orbital is taken over by the (d_*z*^2^_ – π_y_*) orbital: the occupation number of the former orbital is exactly 1, which indicates that it does not contribute to the electron correlation. The discrepancies from the formal uncorrelated values of 2 and 0 are also larger than those of the orbitals providing dynamic correlation only, *e.g.* the π, π* pair of indazole (π_Ind_ and π_Ind_*) (1.93/1.94 and 0.07). Not surprisingly, similar behaviour has been found in {FeNO}^7^ complexes before,^13^ although the effects are even more pronounced there – with occupation numbers of antibonding orbitals as large as 0.3.

**Fig. 4:**
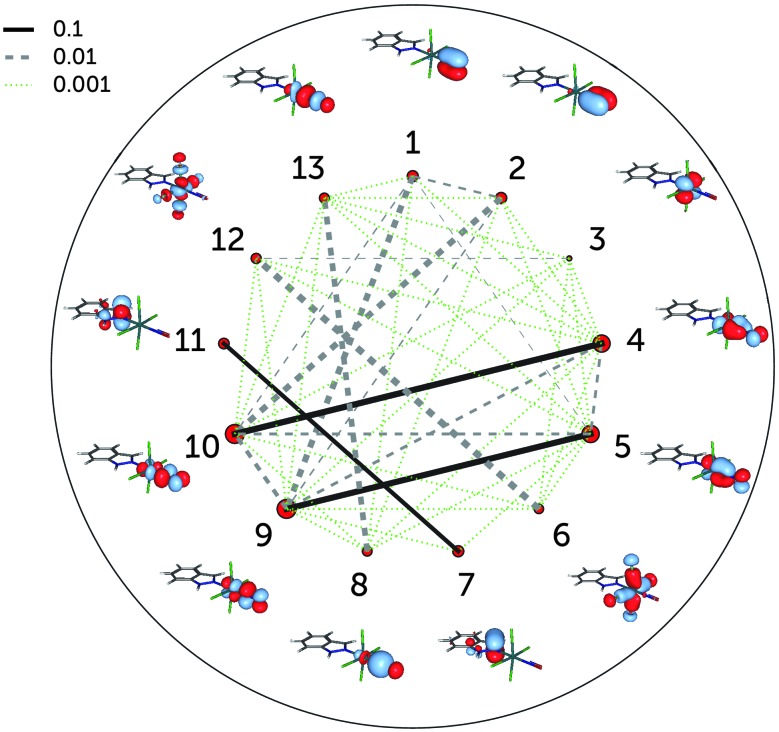
Single-orbital entropy, *s*(1), and mutual information, *I*, in the DMRG(16,13)[1000] (equivalent to the CASSCF) wavefunction of RuHIndNO. The size of the red circles next to the orbitals correlates with the magnitude of the corresponding single-orbital entropy. The lines connecting the dots represent the mutual information: solid lines indicate strong entanglement (*I* > 0.1), dashed grey lines stand for middle entanglement (0.01 > *I* > 0.1) and dotted green lines indicate weak entanglement (0.001 > *I* > 0.01). The line width is also proportional to the absolute value of *I*.

Fig. 4 shows the single-orbital entropies and mutual information for the S_0_ structure, as defined in eqn (1)–(3), as obtained from the DMRG(16,13)[1000] calculation. One can immediately recognise that orbitals 4, 5, 9 and 10 (corresponding to the d_*xz*,*yz*_ ± π_*x*,y_* orbitals) have the largest single-orbital entropy (as indicated by the size of the corresponding red circles in Fig. 4), while *e.g.* orbital 3 (d_*xy*_) shows very low entropy. Orbitals 4, 5, 9 and 10 also show high entanglement with each other, and additionally 9 and 10 are also entangled with the π_*x*,*y*_ orbitals, labelled 1 and 2. Large single-orbital entropies and strong entanglement with more than one orbital are a signature of static correlation. In contrast, small single-orbital entropies combined with weak entanglement among many orbitals or strong entanglement between two orbitals only is an indication of dynamic correlation. Accordingly, the π_*x*,*y*_ – d_*xz*,*yz*_ – π_*x*,y_* orbitals (1, 2, 4, 5, 9, 10), corresponding to two Ru–NO π bonds, are strongly entangled (*i.e.* interact strongly) and are responsible for static correlation. The entanglement of the orbitals 1 with 9 and 2 with 10 is due to dynamic correlation, as expected from ππ* pairs. One can distinguish other orbital pairs which show largely dynamic correlation, *i.e.* have smaller single-orbital entropy and are strongly entangled only with each other, but not with other orbitals of the active space, such as orbitals 7 and 11 (π_Ind_ and π_Ind_*, which are again a textbook example of dynamic correlation), 6 and 12 (d_*x*^2^–*y*^2^_ ± σ_Cl_) and 8 and 13 (d_*z*^2^_ ± σ). The latter two orbital pairs correspond to Ru bonds with chlorido ligands and the Ru–NO σ bond. The single-orbital entropy values correlate well with the deviation of the occupation numbers from 2 or 0 (recall Fig. 1a). The orbitals with the largest deviation (4, 5, 9, 10) show both static and dynamic correlation, whereas orbitals with smaller deviations (7 and 11, 6 and 12, 8 and 13) show mostly dynamic correlation.

**Fig. 5:**
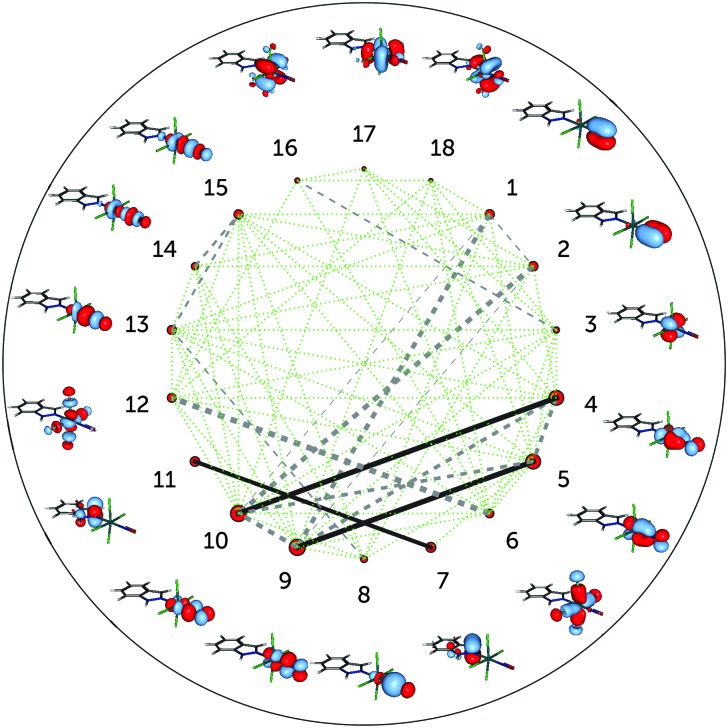
Single-orbital entropy, *s*(1), and mutual information, *I*, for the DMRG(18,18)[1000] wavefunction. Labels as in Fig. 4; additional orbitals have been labelled 14–18.

The incorporation of the additional d_*z*^2^_–NO σ* pair and the double-shell d orbitals in the active space (DMRG(18,18)[1000] calculation) does not change the entanglement picture (Fig. 5). Compared to DMRG(16,13)[1000], only a few weak interactions with the newly added orbitals can be seen. The d_*z*^2^_–NO σ pair (orbitals 8 and 13) has similar single-orbital entropies to the newly added d_*z*^2^_–NO σ* orbitals (14 and 15) and is weakly-entangled with them; similarly weak is the interaction of the d_*xy*_ orbital (3) with its double shell (16). The entanglement of the two other double shells is even smaller – they are not affecting the entanglement in the Ru–NO bond in any way. Single-orbital entropies of other orbitals, present in the smaller active space, remain also unaffected. The few additional weak interactions added with the extension of the active space thus should be attributed to the dynamic correlation and do not affect the overall entanglement picture of the Ru coordination sphere found with the smaller (16,13) active space. The lack of strong entanglement and small single-orbital entropies of the double-shell d orbitals shows that their overall effect on the correlation is negligible, similar to what has been found by Pierloot and coworkers for the description of electronic excitations in other 4d transition metals.^30,46^ The negligible effect of the double-shell d orbitals also explains why the orbital optimisation of the DMRG(18,18)[512]-SCF calculation could only be done with the small ANO-RCC-MB basis set, which excludes the additional p shells of the N, C and O atoms. If the larger ANO-RCC-VTZP basis set is used, the double-shell d orbitals will then be replaced by these p shells. Notably, a similar problem was faced in an earlier study of a ruthenocene complex reported by Phung *et al.* ^61^ We thus emphasise that despite the difference in the basis set, the entanglement picture is similar in both cases.

Summarising, the configurational analysis in terms of CASSCF natural orbitals and the entanglement analyses reveal that the Ru–NO σ bond and other Ru–ligand bonds exhibit mostly dynamic correlation and therefore they are well described with single-configurational methods, whereas the Ru–NO π bonds show static correlation and therefore require a multiconfigurational treatment. It is precisely the electronic structure of these π bonds that contributes to the non-innocence of the NO ligand and, hence, a multiconfigurational analysis is best suited to determine the electronic structure of the {RuNO}^6^ moiety.

An attempt to determine the electronic configuration of Ru can be performed with the help of Mulliken population analysis. For illustrative purposes and for the sake of comparison with the DFT work of Bučinský *et al.*,^4^ we contrast Mulliken population differences of Ru 4d orbitals from wavefunctions obtained from CASSCF and DFT calculations. The total Ru 4d atomic orbital population of the single-determinantal wavefunction is 6.47, in very good agreement with the total Ru d population of 6.54 obtained in a similar Ru nitrosyl complex by Bučinský *et al.* ^4^ The value obtained from the CASSCF wavefunction is 6.02. Given that a single-determinant wavefunction corresponds to a Ru^II^(d^6^)–NO^+^ configuration, *i.e.* to a theoretical Ru 4d occupation of 6, the Mulliken population analysis “overestimates” the d population by approximately half an electron (0.47). Assuming that the intrinsic overestimation of the Mulliken analysis is the same for both wavefunctions, a corrected Mulliken 4d population for the CASSCF wavefunction can be estimated to be 6.02 – 0.47 = 5.55 electrons. A d population value below 6 within a multiconfigurational calculation is due to the mixture of Ru^II^(d^6^)–NO^+^ and Ru^III^(d^5^)–NO^0^ configurations in the wavefunction. Although these values should be considered purely qualitative, the difference in Mulliken populations between single-determinant and the CASSCF wavefunction hints to the need of a multiconfiguration treatment.

Further insight into the Ru coordination sphere can be gained from transforming the CSFs into the basis of localised orbitals (Fig. 2) and analysing the CASSCF wavefunctions in terms of the transformed CSFs. The S_0_ wavefunction expressed in terms of localised orbitals results in a very diffuse expansion, with a large amount of configurations having small but comparable weights, none above 6%. The configurations with the highest weights of 6% and 4% are (σ_Cl_)^2^(d_*xy*_)^2^(d_*yz*_)^2^(d_*xz*_)^1^(d_*z*^2^_)^0^(d_*x*^2^–*y*^2^_)^0^(π_Ind_)^1^(π_Ind_*)^1^(σ_NO_)^2^(π_*x*_)^2^(π_*y*_)^2^(π_x_*)^1^(π_y_*)^0^ and(σ_Cl_)^2^(d_*xy*_)^2^(d_*yz*_)^1^(d_*xz*_)^2^(d_*z*^2^_)^0^(d_*x*^2^–*y*^2^_)^0^(π_Ind_)^1^(π_Ind_*)^1^(σ_NO_)^2^(π_*x*_)^2^(π_*y*_)^2^(π_x_*)^0^(π_y_*)^1^and include the d_*xz*_ → π_x_* and d_*yz*_ → π_y_* excitation, respectively, reflecting two d → π_NO_* back dative bonds along both the *x* and *y* axes. Both of these configurations feature five electrons in Ru d orbitals and five electrons in the NO orbitals, which corresponds to a Ru^III^(d^5^)–NO^0^ character. The configuration with the next-largest contribution (3%) is of a Ru^II^(d^6^)–NO^+^ character:(σ_Cl_)^2^(d_*xy*_)^2^(d_*yz*_)^2^(d_*xz*_)^2^(d_*z*^2^_)^0^(d_*x*^2^–*y*^2^_)^0^(π_Ind_)^1^(π_Ind_*)^1^(σ_NO_)^2^(π_*x*_)^2^(π_*y*_)^2^(π_x_*)^0^(π_y_*)^0^Due to the large number of contributing configurations, a detailed analysis of the character and contributions to the total wavefunction of each particular configuration is not feasible. Instead, we resort to calculating the collective weights of the configurations corresponding to the particular resonance structure. But rather than calculating only weights of *e.g.* Ru^II^–NO^+^ to Ru^III^–NO^0^ configurations (as done in the previous work of Radoń *et al.* ^13^), we classify the CSFs into several classes based on the occupancy of either Ru or ligands, or the collective occupation of Ru and some ligands. The relative weights of configurations belonging to each class are shown in Table 1: in the first class, we consider only the occupation of Ru d orbitals (*cf.* Fig. 2a and Table 1a), then only the NO orbital occupancy (Fig. 2b and Table 1b) equal to *n* = 6 (NO^–^), 5 (NO^0^) or 4 (NO^+^), and finally the combined Ru d and NO occupancy (Table 1c).

**Table 1:** Contributions of all configurations in the S_0_ and T_1_ states with certain properties: (a) only Ru electron configuration taken into account; (b) Only NO electron configuration taken into account; (c) both Ru and NO electron configurations are taken into account; (d) the charge transfer from Cl to NO and Ru is considered

	Character	Contr. S_0_ (%)	Contr. T_1_ (%)
(a)	Ru^IV^(d^4^)	7.7	8.9
	Ru^III^(d^5^)	42.0	44.9
	Ru^II^(d^6^)	39.9	38.4

(b)	NO^–^	18.3	23.1
	NO^0^	58.0	62.3
	NO^+^	21.5	12.2

(c)	Ru^IV^(d^4^) and NO^–^	7.3	8.2
	Ru^III^(d^5^) and NO^0^	31.9	31.2
	Ru^II^(d^6^) and NO^+^	14.3	8.8
	Ru^II^(d^6^) and NO^0^	24.7	28.7

(d)	Ru^II^(d^6^) and (σ_Cl_)^1^	24.6	28.7
	NO^–^ and (σ_Cl_)^1^	10.0	13.2

From the analysis of Table 1a we see that the contribution of Ru^II^(d^6^) and Ru^III^(d^5^) configurations to the S_0_ state is almost the same, yielding a formal oxidation state of Ru of 2.5. This value is in accordance with the corrected Mulliken d population in the CASSCF wavefunction of 5.55 determined previously, despite the fact that Mulliken populations should be treated only qualitatively. A similar process is carried out with NO (Table 1b). The Ru^II^ to Ru^III^ ratios do not correspond to the ratios of ionic to neutral NO: the NO^0^ contribution is the predominant one in this complex (58%). Moreover, NO^+^ contributions are largely cancelled out by NO^–^ contributions.

Since the net charge (–1) of the complex cannot be explained with a Ru formal oxidation state of 2.5 and a NO^0^ ligand, we also consider the class of configurations combining the Ru and NO occupancies. Table 1c shows that the Ru^III^–NO^0^ configurations have the largest collective weight, above 30%, which is 2.2 times as large as that of the Ru^II^–NO^+^ configurations. This weight ratio is slightly smaller than the weight ratio of NO^0^ to NO^+^ configurations, which is approximately 2.7. This shows that the Ru–NO bond situation is dominated by a strong d → π_NO_* back donation leading to NO^0^ and the d^6^ character of Ru comes from elsewhere. Indeed, we find a large amount of configurations with Ru^II^(d^6^) and NO^0^ character, with an even larger weight than that of the Ru^II^(d^6^) and NO^+^ configurations. Table 1d reveals that these configurations entirely correspond to the σ_Cl_ → d_*x*^2^–*y*^2^_ excitations, *i.e.* to a charge transfer from Cl ligands.

Summarising the configuration analysis for the S_0_ state, we may conclude that the electronic structure of {RuNO}^6^ is a mixture of several important contributions from which the Ru^III^–NO^0^ configurations are most important, indicating a strong d → π_NO_* back donation. The Ru^II^–NO^+^ configurations are about half as important if compared by total contributions to the wavefunction, and give Ru some of its Ru^II^ character. Despite the lesser significance of Ru^II^–NO^+^ configurations, Ru shows a large amount of Ru^II^ character, almost equal to its Ru^III^ character by having a formal oxidation state of 2.5. A larger amount of the d^6^ character of Ru, however, does not arise from these configurations, but rather from an electron transfer from the Cl ligands, which can be seen from the contribution of Ru^II^(d^6^)–(σ_Cl_)^1^ configurations: this contribution is almost identical to that of Ru^II^(d^6^)–NO^0^.

Very similar results are obtained for the T_1_ state, despite its different molecular structure and electronic wavefunction. Most notably, the weights of Ru d^5^ and d^6^ configurations are alike and hence the formal oxidation state of Ru is also approximately 2.5. The CSFs with the largest weight have the same electronic configurations as in the case of S_0_, albeit with a different spin and weights under 3%. At first glance, this is quite unexpected since the T_1_ state involves an excitation to a metal–NO π* antibonding orbital and Ru–NO back donation gets stronger. As such one would expect a withdrawal of electron density from the metal to NO. Indeed, we observe it to some extent, as the weight of NO^+^ and Ru^II^(d^6^)–NO^+^ configurations decreases compared to the S_0_ state (8.8% in T_1_ *vs.* 14.3% in S_0_): the bent-coordinated NO has even less NO^+^ contributions than the linear-coordinated one. However, this electron withdrawal from the metal is compensated by the stronger Ru → Cl dative bond: the cumulative weight of Ru^II^(d^6^)–(σ_Cl_)^1^ configurations increases to 28.7%. This stronger dative bond compensates for the electron density loss on Ru due to a stronger back donation, yielding a similar Ru electronic configuration to that in S_0_.

## 4 Conclusion

In this work, we have employed multiconfigurational methods to analyse the electronic structure of the lowest singlet and triplet states of RuHIndNO, a ruthenium nitrosyl complex. We performed a CASSCF calculation on the optimised geometries for the S_0_ and T_1_ states of RuHIndNO and analysed the resulting wavefunction both in terms of CASSCF natural orbitals and localised orbitals. The Ru–NO bond shows strong electronic correlation, both static and dynamic, which is supported by the weight of the dominant configuration being significantly below 100%, comparably large weights of single excitations and large fractional populations of the orbitals involved in this bond and the analysis of orbital entanglement. An orbital entanglement analysis based on the one- and two-orbital reduced density matrices calculated from the DMRG wavefunction of the S_0_ state provides further evidence of strong static correlation in the Ru–NO π bonds, while the Ru–NO σ bond and other Ru–ligand bonds are largely dominated by dynamic correlation. An additional entanglement analysis based on a larger active-space calculation shows a negligible effect of the double-shell d orbitals on the static correlation effects. Furthermore, Mulliken Ru d orbital population based on the single-reference DFT and CASSCF wavefunction shows a discrepancy of approximately 0.5 electrons. In view of these results, we advocate the usage of multiconfigurational methods such as CASSCF to describe the correct bonding situation in the Ru–NO moiety.

CASSCF-type methods also allow for an extensive electronic structure analysis of the metal centre, ligands and metal-ligand bonds in the Ru coordination sphere. By a comparatively straightforward unitary transformation of the active-space orbitals, an operation which does not change the physical content of the wavefunction, we obtain a possibility to quantify the contributions from different electronic configurations and therefore to describe the electronic structure of {RuNO}^6^ more precisely than any assigned formal oxidation state would do. As we have shown, a single structure *e.g.* Ru^III^–NO^0^ or Ru^II^–NO^+^ does not account for the complexity of the {RuNO}^6^ electronic structure. Although the electronic structure of the RuHIndNO complex is a superposition of configurations like Ru^III^–NO^0^, Ru^II^–NO^+^ and many others, we gain more details about the structure when we describe the Ru and NO fragments of the Ru–NO bond separately. In this view, our results show that the electronic configuration of Ru consists of approximately equal amounts of d^5^(Ru^III^) and d^6^(Ru^II^) contributions, resulting in a formal Ru oxidation state of 2.5. The NO electronic configuration, on the other hand, shows a predominantly neutral character, which is in contrast to the commonly accepted picture of the Ru^II^–NO^+^ description. The NO neutral character arises mainly due to the d → π_NO_* back donation, but a dative contribution by the Cl ligands compensates the outflux of the electron density due to this back donation. This description of Ru and NO is almost the same for both the S_0_ and T_1_ state, despite the different electronic structures, with the only difference that metal → NO back donation is even stronger in the T_1_ structure. This increase of the metal → NO back donation is additionally supported by the increase of the bond length in the triplet state.
